# Treatment Strategy for Long COVID Based on Traditional Chinese Medicine

**DOI:** 10.14789/ejmj.JMJ24-0041-P

**Published:** 2025-04-03

**Authors:** MAHIKO NAGASE, YUU TAKESHITA, KIYOHIDE TOMOOKA

**Affiliations:** 1 Division of Medical Education, Juntendo University Faculty of Medicine, Tokyo, Japan; 1 Division of Medical Education, Juntendo University Faculty of Medicine, Tokyo, Japan; 2 Kichijyoji Traditional Chinese Medicine Clinic, Tokyo, Japan; 2 Kichijyoji Traditional Chinese Medicine Clinic, Tokyo, Japan; 3 Hokushin-kai; Academic Society of Traditional Japanese Acupuncture and Moxibustion, Osaka, Japan; 3 Hokushin-kai; Academic Society of Traditional Japanese Acupuncture and Moxibustion, Osaka, Japan; 4 Acupuncture Clinic, Seimei-in, Tokyo, Japan; 4 Acupuncture Clinic, Seimei-in, Tokyo, Japan; 5 Department of Public Health, Juntendo University Faculty of Medicine, Tokyo, Japan; 5 Department of Public Health, Juntendo University Faculty of Medicine, Tokyo, Japan

**Keywords:** long COVID, traditional Chinese medicine, traditional Chinese herbal medicine, Kampo medicine, complementary and alternative medicine

## Abstract

Post-acute COVID-19 conditions, commonly known as ‘long COVID’, occur in individuals with a history of probable or confirmed severe acute respiratory syndrome coronavirus 2 (SARS CoV-2) infection, typically 3 months from the onset of COVID-19 with symptoms, lasting for at least 2 months, that cannot be explained by any alternative diagnoses.

Although most symptoms improve over time, some patients experience significant limitations in their social lives. Under such circumstances, complementary and alternative medicine (CAM) interventions are growing in popularity as possible treatments for long COVID symptoms. There are a wide variety of CAM interventions, such as traditional Japanese medicine (Kampo medicine) and traditional Chinese medicine (TCM). In this study, we compared three studies using Kampo medicine with those using TCM and concluded that Kampo medicine, which is often practiced in Japan due to its historical background, may have some limitations in managing the diverse symptoms of long COVID because the symptoms of long COVID vary widely. Therefore, we propose that medical examinations based on TCM should also be considered as an alternative treatment strategy for patients with long COVID.

## Introduction

Post-acute COVID-19 conditions, commonly known as ‘long COVID’, occur in individuals with a history of probable or confirmed SARS CoV-2 infection, typically 3 months from the onset of COVID-19 with symptoms, lasting for at least 2 months, that cannot be explained by any alternative diagnoses^1)^. Symptoms of long COVID may be new-onset following initial recovery from an acute COVID-19 episode or persist from the initial illness, and they may also fluctuate or relapse over time^2)^. Common symptoms include: fatigue, shortness of breath, and cognitive dysfunction, all of which generally affect daily functioning. Even with the World Health Organization (WHO) ending the global emergency status for COVID-19 in May 2023, with over 600 million cases globally, the burden of disease attributable to post-acute COVID-19 remains a major public health issue^3)^. To date, hundreds of biomedical findings have been documented, with many patients experiencing dozens of symptoms of long COVID involving multiple organ systems. However, long COVID symptoms are still largely unknown, and no standard treatment has been established. Although most symptoms improve over time, some patients experience significant limitations in their social lives. Therefore, it is important to respond from a multifaceted and holistic perspective because of the wide variety of symptoms and processes involved^4)-5)^. Under such circumstances, complementary and alternative medicine (CAM) interventions are growing in popularity as possible treatments for long COVID symptoms. Recently, it was reported that CAM interventions may be effective, safe, and acceptable for patients with such symptoms^6)^. There are wide variety of CAM interventions, one of which is traditional Chinese medicine (TCM). TCM is a complete system of healing that was developed in China about 3,000 years ago. It includes herbal medicine and acupuncture as two of the most important components of CAM and plays a key role in the realization of integrative medicine. In recent decades, the use of TCM has become more popular in China and throughout the world^7)^. Traditional Japanese medicine (Kampo medicine) has been used for 1,500 years, including Kampo-yaku (herbal medicine) and acupuncture in Japan. Kampo medicine is now widely practiced in Japan and has become fully integrated into the modern health-care system. It is based on TCM but has been adapted to complement Japanese medical situations^8)^. In this study, we aim to examine the treatment strategy for long COVID in Japan by comparing studies using Kampo medicine with those using TCM.

## Effective cases of long COVID treatment using Kampo medicine

Harada et al. reported two cases of hair loss due to coronavirus disease 2019 (COVID-19), sequelae of long COVID, successfully treated with ninjin’ yoeito. They administered ninjin’ yoeito for patterns of both qi and blood deficiency with reference to traditional key treatment, and concluded that it contributed to the improvement of symptoms^9)^. Ono et al. reported that fatigue after COVID-19 may be improved by saikokeishito, especially for the treatment of patients with slight fever or inflammatory findings^10)^. In addition, Tokumasu et al. reported that analysis of clinical data from 102 long COVID patients with general fatigue and accompanying symptoms revealed that the patients had various symptoms, and the most frequent ones accompanying general fatigue were: dysosmia, dysgeusia, headache, insomnia, dyspnea, and hair loss. In that study, Kampo medicine accounted for 24.1% of the total prescriptions (n = 609). The most frequently prescribed Kampo medicine was hochuekkito (71.6%); other prescribed Kampo medicines were tokishakuyakusan, ryokeijutsukanto, juzentaihoto, hangekobokuto, kakkonto, ninjin’yoeito, goreisan, rikkunshito, and keishibukuryogan^11)^. Based on these results, one single type of Kampo-yaku (herbal medicine) tends to be administered for each patient in Japanese trials.

## Effective treatment of long COVID using traditional Chinese medicine

Fu et al. reported a case of brain fog, one of the more common long COVID symptoms, for which they administered a combination-drug consisting of bu zhong yi qi tang, wen dan tang, wu wei xiao du yin ban lan gen, da qing ye, and chuan xin lian and the patient improved^12)^. Hsieh et al. reported that personalized traditional Chinese herbal medicine notably reduced fatigue as well as respiratory and emotional distress symptoms after 14- and 28-day treatment in patients with COVID-19 sequelae. In that study, they administered 27 types of traditional Chinese herbal medicine formulas and single herbs^13)^. Furthermore, Zhong et al. evaluated the effects of TCM on the health conditions of post-COVID-19 patients, particularly with the traditional Chinese medicine syndrome diagnosis and body constitutions (an inherent trait of the human body in morphological structure, physiological function and psychological status on the basis of innate inheritance and acquired acquisition, which determines the susceptibility of individuals to certain diseases and similarities in pathological processes^14)^) as well as related clinical characteristics^15)^. In that clinical trial, fever, fatigue, and dry cough were the most common symptoms, present in 59.3, 55.3, and 46% of participants, respectively. Among the 150 post-COVID patients, the majority (71.3%) had one of two post-COVID TCM syndromes (qi deficiency of lung and spleen, and qi and yin deficiency). The participants received various types of combined TCM treatment based on the recommended prescriptions in the TCM clinical practice guidelines for COVID-19 patients, individual TCM syndromes, and clinical symptoms for three to six months. Upon TCM treatment, there was an observable increase in participants achieving a balanced body constitutions (i.e., healthy body conditions). The increase was more prominent in those without compared with TCM syndromes. The main clinical symptoms in participants with TCM syndromes decreased upon TCM treatment. In summary, as compared with Kampo medicine trials in Japan, clinical studies in TCM commonly involve combining multiple prescriptions, such as 2-3 different formulas, within a single treatment period for each patient.

## Discussion

The WHO defined long COVID as the continuation or development of new symptoms 3 months after the initial SARS-CoV-2 infection, with these symptoms lasting for at least 2 months in the absence of any other explanation^16)^. Recently, a meta-analysis revealed that more than 50 symptoms have been identified in long COVID patients, approximately 80% of whom suffer from one or more symptoms^17)^. As possible causes of these symptoms, multiple etiologies such as direct organ damage, constitutive inflammatory responses including autoimmunity, and psychiatric impairment may be involved^18)^.

Under such circumstances, CAM interventions including Kampo medicine and TCM are growing in popularity as possible treatments. Kampo medicine is based on TCM but is adapted to Japanese culture. It can be characterized as a simplified version of TCM^19)^. The process of diagnosis and treatment differs between TCM and Kampo medicine. Kampo methodology for diagnosis and treatment is referred to as "formulation corresponding to sho (Kampo diagnosis); each pathologic condition is thus related to its prescription. For example, diseases that are expected to respond to kakkonto therapy are diagnosed as kakkonto-sho and it naturally follows that kakkonto is prescribed in such cases^20)^. The relationship between these steps is regarded as similar to that of a lock and key^21)^; therefore, only one type of Kampo-yaku (herbal medicine) tends to be administered to each patient. On the other hand, in TCM, after having recognized the particular zheng (TCM diagnosis), the Chinese practitioner then confirms the ‘treatment principle’. According to this principle, it is possible to choose formulae for treatment and adjust the herbs used in the formula, or make a new formulation adapted to the patient’s particular condition. This step is called the ‘selection of treatment’ in TCM. The process from diagnosis to treatment in TCM is called the ‘selection of treatment based on the differential diagnosis’^7)^ Thus, the use of a combination of two or three herbal medicines for each patient has been proposed as effective intervention against long COVID from the perspective of TCM, because residual symptoms of COVID-19 are often viewed as a manifestation of the imbalance of various kinds of TCM syndrome diagnosis and body constitutions^22)^. In fact, a multi-center observational study in Hong Kong showed that individualized TCM treatments could help resolve clinical symptoms (including fatigue, cough, shortness of breath, post-meal fullness, and loose stools), improve lung functions, and lead to healthier TCM body constitutions in COVID-recovered patients^14)^. Recently, we reported that long COVID symptoms were not uniform, with malaise (17%) and cough (15%) being slightly more frequent (31 types), and the 46 herbal medicines prescribed also varied^23)^. The most frequently prescribed Chinese herbal medicines were toukishakuyakusan and bakumondoutou, each accounting for about 6.8% of all prescriptions. In our study, the most common body constitutions associated with fatigue were blood deficiency and water retention, which are indications for toukishakuyakusan. In addition, lung yin deficiency, an indication for bakumondoutou, is frequently noted in the presence of coughing^20)^. The symptoms of long COVID vary widely; thus, we can conclude that it is important to prescribe a combination of TCM on an understanding of the pathophysiology of the disease to improve symptoms. Furthermore, Chen et al. reported that compared to Western medicine treatment, TCM treatment effectively improved clinical symptoms and shortened the duration of symptoms in 1426 cases with long COVID^24)^. With these considerations, although Kampo medicine is often practiced in Japan due to its historical background, it may have some limitations in managing the diverse symptoms of long COVID. Hence, we suggest that incorporating medical examinations grounded in TCM could also serve as a viable intervention for individuals experiencing long COVID.

## Conclusion

The diagnostic approaches and examination techniques of TCM are distinct from those used in Japanese Kampo medicine. Consequently, integrating these methods could offer potential relief for symptoms associated with long COVID. Additionally, mitigating long COVID symptoms and reducing the treatment duration may contribute positively to public health, particularly in managing workforce well-being.

## Funding

No funding was received.

## Author contributions

MN wrote the manuscript. YT and KT read and approved the final manuscript.

## Conflicts of interest statement

MN receive lecture fee from Tsumura & Co.
